# Next-generation genotyping of hypervariable loci in many individuals of a non-model species: technical and theoretical implications

**DOI:** 10.1186/s12864-016-2503-y

**Published:** 2016-03-08

**Authors:** Kathleen E. Grogan, Gwendolyn J. McGinnis, Michelle L. Sauther, Frank P. Cuozzo, Christine M. Drea

**Affiliations:** University Program in Ecology, Duke University, Durham, NC USA; Department of Evolutionary Anthropology, Duke University, Durham, NC USA; Department of Anthropology, University of Colorado-Boulder, Boulder, CO USA; Department of Anthropology, University of North Dakota, Grand Forks, ND USA; Department of Biology, Duke University, Durham, USA; Emory University, Room 2006 O. Wayne Rollins Research Center, 1510 Clifton Rd NE, Atlanta, GA 30322 USA

**Keywords:** 454 Titanium, Ion Torrent PGM, Major Histocompatibility Complex, Ring-tailed Lemur, *Lemur catta*, Bezà Mahafaly, Madagascar

## Abstract

**Background:**

Across species, diversity at the Major Histocompatibility Complex (MHC) is critical to disease resistance and population health; however, use of MHC diversity to quantify the genetic health of populations has been hampered by the extreme variation found in MHC genes. Next generation sequencing (NGS) technology generates sufficient data to genotype even the most diverse species, but workflows for distinguishing artifacts from alleles are still under development. We used NGS to evaluate the MHC diversity of over 300 captive and wild ring-tailed lemurs (*Lemur catta:* Primates: Mammalia). We modified a published workflow to address errors that arise from deep sequencing individuals and tested for evidence of selection at the most diverse MHC genes.

**Results:**

In addition to evaluating the accuracy of 454 Titanium and Ion Torrent PGM for genotyping large populations at hypervariable genes, we suggested modifications to improve current methods of allele calling. Using these modifications, we genotyped 302 out of 319 individuals, obtaining an average sequencing depth of over 1000 reads per amplicon. We identified 55 MHC-*DRB* alleles, 51 of which were previously undescribed, and provide the first sequences of five additional MHC genes: *DOA*, *DOB*, *DPA*, *DQA*, and *DRA*. The additional five MHC genes had one or two alleles each with little sequence variation; however, the 55 MHC-*DRB* alleles showed a high dN/dS ratio and trans-species polymorphism, indicating a history of positive selection. Because each individual possessed 1–7 MHC-*DRB* alleles, we suggest that ring-tailed lemurs have four, putatively functional, MHC-*DRB* copies.

**Conclusions:**

In the future, accurate genotyping methods for NGS data will be critical to assessing genetic variation in non-model species. We recommend that future NGS studies increase the proportion of replicated samples, both within and across platforms, particularly for hypervariable genes like the MHC. Quantifying MHC diversity within non-model species is the first step to assessing the relationship of genetic diversity at functional loci to individual fitness and population viability. Owing to MHC-*DRB* diversity and copy number, ring-tailed lemurs may serve as an ideal model for estimating the interaction between genetic diversity, fitness, and environment, especially regarding endangered species.

**Electronic supplementary material:**

The online version of this article (doi:10.1186/s12864-016-2503-y) contains supplementary material, which is available to authorized users.

## Background

In the era of DNA sequencing, researchers must balance cost, ease of use, efficiency, sequencing fidelity, and quantity of data produced against project requirements when choosing between sequencing technologies. Despite the rapid proliferation of next generation sequencing (NGS) technologies, we have relatively few evaluations of the accuracy and efficiency of NGS platforms for whole genome sequencing or for re-sequencing of large populations [1–5]. Here, we genotype 319 ring-tailed lemurs (*Lemur catta:* Primates: Mammalia) at the Major Histocompatibility Complex (MHC) gene *DRB*, using two NGS platforms, Roche's 454 FLX Titanium (Nutley, NJ, USA) and Life Technology's Ion Torrent Personal Genome Machine (PGM). We demonstrate the utility of using NGS to genotype non-model organisms at complex hypervariable loci like those of the MHC and we validate the use of MHC-*DRB* diversity as a proxy for overall MHC diversity.

MHC genes are among the most variable in the mammalian genome, presumably because of their role in identifying the myriad potential pathogens in the environment [6]. MHC protein products activate the adaptive immune system whenever they encounter and bind an intracellular or extracellular pathogen. Across fish, reptiles, birds, and mammals, MHC genes are frequently duplicated with 1–9 copies and 1–1,400 alleles per gene: Even between alleles within a single species, 12-45 % of the nucleotide sites are variable [6–21]. Although this diversity and genomic complexity makes the MHC well suited for the measurement of individual and population-level genetic fitness, it leads to many practical complications during MHC genotyping [22–24]. Beyond the difficulties introduced by duplications, allelic variation, and the presence of pseudogenes (reviewed in [25]), genotyping population-level sample sizes (> 50 individuals) for studies of the influence of MHC on health or reproductive success can be cumbersome, time-consuming, and expensive.

Although many techniques have been used to genotype the MHC (reviewed in [25]), cloning has historically been the gold standard [22, 25, 26]; however, MHC allelic variation and duplication can necessitate higher sequence coverage than is practical for cloning or gel-based genotyping systems. Parallel sequencing using NGS platforms solves this problem by quickly generating immense volumes of data [27, 28]. Moreover, in studies comparing the performance of NGS platforms to cloning or gel-based genotyping systems, NGS detects significantly more MHC alleles [19, 29, 30]. To overcome the difficulty of genotyping hypervariable loci for large sample sizes, samples can be pooled or multiplexed into a single run via parallel-tagged sequencing on NGS platforms [27, 31, 32]. NGS technology is, therefore, less labor intensive, more efficient, and more cost effective than older methods. A significant challenge with these technologies, however, is distinguishing genuine allelic variation from artifacts.

Current NGS platforms include GS FLX Titanium/GS FLX Junior from Roche, SOLiD and Ion Torrent platforms from Life Sciences, the PacBio RS II from Pacific Biosciences, and Genome Analyzer/HiSeq/MiSeq/NextSeq from Illumina. Owing to the specifics of enzymology, chemistry, high-resolution optics, hardware, and software, each platform differs in the types of sequencing errors (i.e., artifacts) (reviewed in [33–35]) and quantity of data produced (for detailed methodology, see [1, 3–5, 36–44]). Most NGS projects sequence genomes via the assembly of short (60–150 base pair or bp) reads. As NGS platforms became cheaper and produced longer reads [45], however, ecologists and conservation biologists began using NGS to obtain population-level estimates of genetic parameters and individual genotypes for hundreds or thousands of samples [19, 27, 29–32]. Currently, Roche 454 FLX, Ion Torrent PGM, PacBio RS, and Illumina MiSeq v3 are the only NGS platforms to rival Sanger sequencing for the production of longer (>400 bp) reads [45]. Roche 454 was the first platform to produce longer reads and thus has been most widely used; however, because Roche is shutting down the 454 FLX manufacturing and servicing at the end of 2016, it is critical to evaluate the performance of this and other NGS technologies against traditional methods like cloning, as well as to be able to distinguish alleles from sequencing errors or artifacts.

In this paper, we use both the Ion Torrent 314 v2 chip and 400 bp kit, as well as the 454 FLX Titanium, to genotype the second exon of the MHC-*DRB* of a non-model species. We present modifications to a method for distinguishing alleles from sequencing errors to account for potential pitfalls of deep sequencing. Additionally, we report on allelic diversity in the ring-tailed lemur, which is only the third strepsirrhine primate species to be investigated in depth [12, 19, 46–50]. In species for which the MHC is well characterized, identifying alleles and assigning genotypes is relatively straightforward; however, for most non-model species, basic information about the number of gene copies and the variability present in the MHC is unavailable. In such cases, distinguishing between true alleles and artifacts presents a substantial methodological challenge because of the extreme variation and copy number variability in MHC genes [19, 21, 51, 52].

Ten years after beginning to use NGS in genotyping, investigators are still developing standards for quality controls, validation, and allele calling (reviewed in [53]). In one strategy, researchers used a cut-off based on the absolute frequency [27, 54, 55] or the relative frequency of alleles and artifacts within an amplicon [51, 56, 57]. Because the sequencing depth often causes the relative frequencies of alleles and artifacts to overlap near the cut-off [58], this latter approach may result in misassignments, including false negatives, in which genuine alleles are discarded as artifacts, and false positives, in which artifacts are classified as alleles [19, 21, 51, 52, 56, 59–63]. Therefore, researchers have begun implementing new strategies (e.g., [64]), custom algorithms (e.g., [58, 59, 65], but see [61]), and workflows to account for differential amplicon efficiency (e.g., [19, 60]). By modifying a published protocol, we present additional steps to refine the process of assigning correct genotypes for the MHC-*DRB* gene.

As our contribution to these workflows, we add steps to address two problems that occur in NGS genotyping during deep sequencing: allelic dropout and misassignment of artifacts. In current allele-calling workflows, researchers classify as an allele any sequence that does not meet the criteria of an artifact. Criteria for an artifact involve filtering by length, quality, consensus to known sequences, presence in sample replicates or in other individuals, and relative frequency within an amplicon. Some of these criteria must be adjusted depending on the depth of coverage and number of amplicons.

Because the second exon of MHC-*DRB* contains the functionally important antigen-binding site, which is responsible for peptide recognition, the MHC-*DRB* gene is one of the best-studied and most diverse functional genes [6, 23]. Researchers often use diversity at the MHC-*DRB* gene as a proxy for diversity across the rest of the MHC gene family (reviewed in [66]); however, this practice may lead to an inaccurate estimation of MHC diversity because the MHC spans hundreds of genes over several megabases of DNA [67, 68]. Our ultimate goal was to compare multiple MHC genes to verify that the MHC-*DRB* gene is the most variable and, therefore, a suitable target for future work investigating MHC contributions to fitness. We evaluated allelic diversity at five additional MHC genes that are typically less diverse in primates than is the MHC-*DRB* (reviewed in [67, 69]). For all six MHC genes, we examined nucleotide and amino acid variation, as well as the presence or absence of selective pressure on MHC-*DRB*, by looking for amino acid sites under positive selection and trans-species polymorphism (reviewed in [70]). As the second exon of MHC-*DRB* encodes the functionally important binding pocket responsible for the specific binding of pathogenic peptides, selection is expected to act most intensely on this part of the gene. Therefore, this area of the MHC has been used as a barometer for the genetic health of populations [23, 71]. Studies like the present one pave the way for using NGS to provide reliable genotyping of hypervariable loci on large numbers of non-model species.

## Methods

### Subjects and sampling

Our study population consisted of 319 ring-tailed lemurs. These represented (a) 126 captive animals from various facilities, including the Duke Lemur Center (DLC) in Durham, NC (*n* = 105), the Cincinnati Zoo in Cincinnati, OH (*n* = 3), and the Indianapolis Zoo in Indianapolis, IN (*n* = 18), as well as (b) 193 wild animals from the Bezà Mahafaly Special Reserve (BMSR) in southwestern Madagascar. For captive animals, our sampling methods for obtaining DNA (see below) followed approved animal handling guidelines and protocols of the Institutional Animal Care and Use Committee of Duke University (most recent Duke University IACUC #A143-12-05, approved 05/25/2012), as well as the institutional guidelines of each zoo. Sample collection from wild lemurs in Madagascar was approved by the Institutional Animal Care and Use Committees of the University of Colorado-Boulder and/or the University of North Dakota (most recent University of North Dakota IACUC #0802-2 approved 04/03/08), Madagascar National Parks (MNP, formerly known as the Association Nationale pour le Gestion des Aires Protégées or ANGAP), and CITES (05US040035/9).

For individuals derived from the captive populations, either staff veterinarians obtained blood samples from the femoral vessels of gently hand-restrained subjects or we acquired tissue samples banked from deceased subjects. These samples were stored at −20 °C until processing. Blood samples from anesthetized, wild animals were obtained by team veterinarians during annual health analyses conducted from 2003–2012 (e.g., [72–76]). The blood samples from 2003–2006 were preserved on Schleicher & Schuell IsoCode^©^ DNA Isolation Cards (*n* = 123; Keene, NH, USA), whereas blood samples from 2007–2012 were preserved on Whatman FTA® Classic cards (*n* = 70; GE Healthcare Life Sciences, Buckinghamshire, UK; [75, 77]).

### DNA extraction and genotyping overview

For samples obtained from captive animals, we performed DNA extractions using either DNA miniprep kits (Sigma, St. Louis, MO, USA) or DNeasy® Blood and Tissue kits (Qiagen, Valencia, CA, USA; [78]). For samples obtained from wild animals, we extracted DNA from the IsoCode cards, using 3.0-mm hole punches following manufacturer's instructions, and from FTA cards following the protocol for Whole Genome Amplification (WGA) of DNA from blood spots dried on FTA paper (Qiagen). Due to the age of some cards and the storage conditions in Madagascar, we subjected each extracted DNA sample to WGA to improve DNA quality and quantity. Previously, researchers have shown that WGA results in 98 % congruence of SNP calling between amplified and non-amplified DNA, and does not result in allelic dropout [79]. We performed WGAs using Repli-G Single Cell Kits® (Qiagen) and modified our protocol by incubating each sample for 16 h at 30 °C to generate sufficient quantities of gDNA for future work. To verify that the WGA did not bias our genotyping results, we also subjected the DNA of a subset of captive individuals (*n* = 36) to WGA. We then genotyped all individuals at the second exon of MHC-*DRB* using the 454 FLX Titanium® (Roche, Nutley, NJ, USA) and the Ion Torrent PGM® (Life Technologies, Grand Island, NY, USA) platforms. For verification of NGS genotyping, we cloned the MHC-*DRB* second exon from 19 captive individuals. We also genotyped 5–10 individuals at five additional MHC loci (including MHC-*DOA*, MHC-*DOB*, MHC-*DPA*, MHC-*DQA*, and MHC-*DRA*) via cloning [16, 80].

### MHC-*DRB* primers

For NGS sequencing, we amplified a 171 bp-fragment of the MHC-*DRB* exon 2 using modified primers, JS1 and JS2 [81]. The 454 forward primer was composed of the 454 FLX amplicon A 19-bp adaptor sequence, a 4-bp key sequence, a 10-bp multiplex identifier ‘tag’ (indicated with Ns), and the site-specific forward primer, JS1 (underlined): 5’ < CGTATCGCCTCCCTCGCGCCATCAGNNNNNNNNNNGAGTGTCATTTCTWCAACGGGACG > 3’. The Ion Torrent forward fusion primer was composed of the Ion Torrent A adaptor sequence, a 4-bp key sequence, a 10-bp multiplex identifier ‘tag’, and the site-specific forward primer, JS1: 5’ < CCATCTCATCCCTGCGTGTCTCCGACTCAGNNNNNNNNNNGATGAGTGTCATTTCTWCAACGGGACG > 3’. The reverse primers also included the platform-specific adaptors, 4-bp key sequence, 10-bp multiplex identifier ‘tag’, and the site-specific reverse primer, JS2. The 454 reverse primer and Ion Torrent reverse primer sequences, respectively, were as follows: 5’ < CGTATCGCCTCCCTCGCGCCATCANNNNNNNNNNGATCCCGTAGTTGTGTCTGCA > 3’ and 5’ < CCTCTCTATGGGCAGTCGGTGATTCAG-NNNNNNNNNNGATGATCCCGTAGTTGTGTCTGCA > 3’. We used 12 distinct 10-bp tags from the standard MID set developed by the manufacturer (Roche, Nutley, NJ, USA).

### 454 Sequencing of MHC-*DRB*

We performed PCRs for 454 sequencing on a programmable iCycler thermocyler (Bio-Rad, Hemel Hempstead, UK) in 25 μL reactions, using 2.5 μL of 10X FastStart High Fidelity Reaction Buffer # 2 with MgCl_2_, 10 μM of each primer, 5 mM of each dNTP, 1.25 U of FastStart High Fidelity Taq Polymerase (Roche, Nutley, NJ, USA), and 20–70 ng of genomic DNA per reaction. The PCR scheme was as follows: initial denaturation at 94 °C for 3 min, 25 cycles of 94 °C for 15 s, 55 °C for 45 s, 72 °C for 1 min, followed by a final extension at 72 °C for 8 min. We estimated the concentration of the PCR products by agarose gel electrophoresis [21, 82] and combined approximately equimolar quantities of each PCR product into five pools. We combined 56–96 unique individuals per pool, plus 19–30 replicates, for a total of 80–115 PCR reactions per pool (Table 1). In total, we pooled 494 PCR reactions (i.e., amplicons), from 319 individuals, sequencing these samples according to the manufacturer’s instructions on five 1/8^th^ lanes of a 454 PTP Titanium plate ([detailed platform methods are (reviewed in 1, [3–5, 36–44])). We sequenced these pooled amplicons between September 2011 and October 2013 at the Genome Sequencing & Analysis Core Resource, Duke University, NC, and the Microbiome Core Facility, University of North Carolina at Chapel Hill, NC.

**Table 1:** NGS statistics per 454 Titanium 1/8^th^ lane or individual Ion Torrent PGM run

Run identity	Initial number of reads generated	Initial number of amplicons pooled [number of replicates]	Reads post filtering on Galaxy [% of initial reads]	Final number of amplicons over read threshold of 120 [number of replicates]	Final mean [and range] of read coverage per amplicon
454 Titanium lane 1	138,185	80 [24]	104,937 [75.9 %]	52 [16]	1,448.5 [120–23,492]
454 Titanium lane 2	98,474	102 [21]	78,642 [79.9 %]	30 [0]	1,841.9 [120–11,048]
454 Titanium lane 3	90,355	102 [30]	38,903 [43.1 %]	101 [29]	299.8 [165–471]
454 Titanium lane 4	103,559	115 [19]	29,769 [28.8 %]	81 [13]	210.7 [120–638]
454 Titanium lane 5	108,867	95 [19]	43,921 [40.3 %]	79 [12]	393.8 [120–1,313]
Ion Torrent run 1	607,318	120 [12]	277,195 [45.6 %]	99 [5]	1,885.6 [120–7,190]
Ion Torrent run 2	573,126	120 [16]	198,604 [34.6 %]	99 [8]	1,415.2 [120–5,727]
Ion Torrent run 3	693,065	121 [14]	341,675 [49.3 %]	101 [5]	2,534.6 [120–15,851]
All Runs	2,176,290	855 [155]	930,067 [42.7 %]	642 [88]	1087.8 [120–23,492]

### Ion torrent PGM sequencing of MHC-*DRB*

We performed PCR reactions for Ion Torrent sequencing on programmable iCycler thermocyler (Bio-Rad, Hemel Hempstead, UK) in 50 μL reactions, with 44 μL Platinum PCR Supermix High Fidelity (Invitrogen, Life Technologies, Grand Island, NY, USA), 10 μM of each primer, and 20–70 ng of genomic DNA. Based on the performance of our samples on the 454 Titanium platform, we performed each PCR as a touchdown series of cycles to decrease the production of PCR artifacts [28]. Initial denaturation began at 94 °C for 2 min, followed by touchdown PCR for 14 cycles: denaturation at 94 °C for 30 s, annealing 62 °C for 30 s, extension at 68 °C for 1 min, and lowering the annealing temperature by 0.5 °C every cycle, ending at an annealing temperature of 55 °C. Following the initial 14 cycles, we performed 30 additional cycles as follows: denaturation at 94 °C for 30 s, annealing at 55 °C for 30 s, extension at 68 °C for 1 min, followed by a final extension at 68 °C for 10 min. We followed the previously described protocol for pooling PCR products into pooled amplicons, and submitted these pools to the Genome Sequencing & Analysis Core Resource at Duke University in September and October of 2013 (for detailed platform methods, see [1, 3–5, 36–44]). For each Ion Torrent PGM run, we pooled 104–108 unique individuals and 12–16 replicates, for a total of 120–121 amplicons per run (Table 1). In total, we pooled and sequenced 361 amplicons of 319 individuals on three Ion Torrent PGM runs. We used the Ion Torrent PGM Template OT2 400 Kit and an Ion Torrent PGM 314R v2 chip for sequencing, and Ion Torrent Software Suite 3.6 for image analysis, according to the manufacturers’ instructions.

### Cloning of MHC genes: *DRB*

To verify NGS genotypes of the second exon of MHC-DRB, we used the unmodified primers JS1 and JS2 to PCR 18 samples following the above Ion Torrent PCR protocol. Following manufacturers’ instructions, we cloned these PCR products using pGEM-T® Easy Vector (Promega, Madison, WI) and Library Efficiency® DH5α Competent Cells (Invitrogen, Life Technologies, Grand Island, NY). Due to the incredible diversity of MHC-DRB, we sequenced between 50 and 90 clones per individual, using ABI 3730xL Analyzer and Big Dye chemistry (Applied Biosystems®, Life Technologies, Grand Island, NY). Using MEGA 5.2 [83], we aligned and analyze sequences against NGS sequences for these individuals.

### Cloning of MHC genes: *DOA*, *DOB*, *DPA*, *DQA*, and *DRA*

To clone additional MHC genes, we designed primers from grey mouse lemur (*Microcebus murinus*), thick-tailed bushbaby (*Otolemur garnetti*), and Philippine tarsier (*Tarsius syrichta*) Genbank sequences (Additional file 1: Table S1). Our PCR had an initial denaturation of 45 s at 94 °C, followed by 30 cycles of 30 s at 94 °C, 30 s at 54 °C, and 1 min at 68 °C, and a final extension of 7 min at 68 °C. Using pGEM-T® Easy Vector (Promega, Madison, WI) and Library Efficiency® DH5α Competent Cells (Invitrogen, Life Technologies, Grand Island, NY), we cloned the PCR products following manufacturers’ instructions. In other primate species, these genes have much reduced diversity compared to MHC-*DRB* [16, 80]. We therefore sequenced only 10–30 positive clones per gene, per individual, on ABI 3730xL Analyzer using Big Dye chemistry (Applied Biosystems®, Life Technologies, Grand Island, NY). We considered as alleles only sequences found in minimally three clones per PCR. We used MEGA 5.2 [83] to align and analyze sequences from MHC-*DOA*, MHC-*DOB*, MHC-*DPA*, MHC-*DQA*, and MHC-*DRA*.

### NGS data analysis

**Fig. 1:**
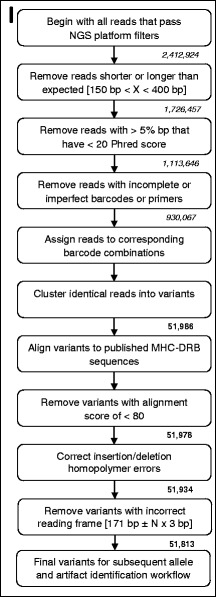
Workflow for differentiating alleles from artifacts: Step I. This workflow is modified from [19] and a more detailed description of the workflow is provided in the Additional file 1. Briefly, NGS reads were filtered for read length, Phred quality score, complete forward and reverse primer + MID, and alignment to published sequences. Read numbers are given in italics, whereas variant numbers are given in bold

**Fig. 2:**
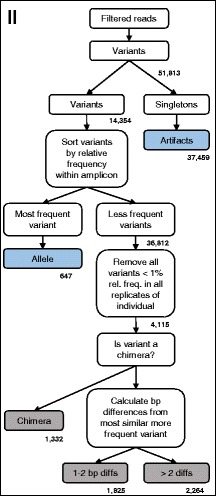
Workflow for differentiating alleles from artifacts: Step II. This workflow is modified from [19] and a more detailed description of the workflow is provided in the Additional file 1. In Step II, identical reads within each amplicon were clustered into a single variant and variants were sorted according to relative frequency within the amplicon. Variants of > 1 % frequency and chimeric variants were discarded. Then variants were classified as differing from the most frequent sequence by 1–2 bp or > 2 bp. Intra-amplicon classifications are shaded in gray, whereas final variant identifications are shaded in blue. Variant numbers are given in bold

**Fig. 3:**
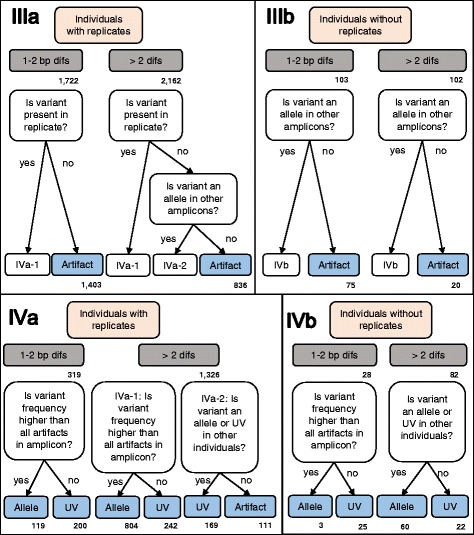
Workflow for differentiating alleles from artifacts: Step III & IV. This workflow is modified from [19] and a more detailed description of the workflow is provided in the Additional file 1. Variants were compared to replicate amplicons for each individual using relative frequncies, and to amplicons of other individuals to determine if the variant was an allele, a low efficiency allele, an artifact, or an unclassified variant. A more detailed description of the workflow is provided in the Additional file 1

**Fig. 4:**
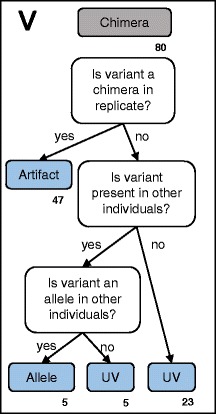
Workflow for differentiating alleles from artifacts: Step V. This workflow is modified from [19] and a more detailed description of the workflow is provided in the Additional file 1. Variants classified as chimeric sequences in Step II were compared to other replicates to determine if they were truly chimeric sequences. A more detailed description of the workflow is provided in the Additional file 1

**Fig. 5:**
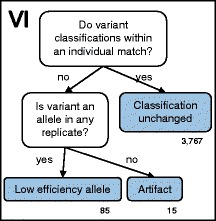
Workflow for differentiating alleles from artifacts: Step VI. This workflow is modified from [19] and a more detailed description of the workflow is provided in the Additional file 1. In the final step of the workflow, we compared the classification of each variant across replicates within an individual to verify that no classifications disagreed

Following initial quality assessment by the 454 and Ion Torrent software, we differentiated alleles from artifacts using a modified version of a published protocol (Figures 1–5; see [19]). In our first step, we used the open-sourced, web-based platform Galaxy to filter the original FASTQ files for read length and read quality [84–86]. We discarded all reads shorter than 150 bp, longer than 400 bp, or in which more than 5 % of bp had a Phred score < 20. We then sorted reads into specific amplicons according to their unique barcode combination using jMHC [87]. Using the Galaxy Clustalw package [88], we aligned reads to published ring-tailed lemur MHC-*DRB* sequences (Genbank: AB078199, AB078201, AB078229, AB078247, AB078248, AB078265, AB078279, AB078287, AB078288, AB078292, AB078301, AB078303; [46]). Because the number of MHC-*DRB* copies present in the ring-tailed lemur was unknown, we initially analyzed all reads assuming that ring-tailed lemurs had only one copy. We also assumed an average amplicon efficiency of 0.70, as outlined in previously published methods [19]. We therefore discarded amplicons containing < 25 reads as having too few reads to genotype accurately [19, 27, 28]. After discarding singleton variants, we compared replicates of the same individual and discarded any variant that did not represent > 1 % of the total proportion of reads in any replicate amplicon; these variants were considered artifacts [31]. We performed all subsequent steps of the workflow (Figs. 1, 2, 3, 4, and 5) independently on each amplicon without regard to results from replicates of the same individual. We performed each step on all amplicons before beginning the next step of the workflow, i.e., we completed Step I for all amplicons before beginning Step II, then completed Step II before moving on to Step III. We analyzed variants within each amplicon independently and did not assume each only had one classification; thus, even if a variant were classified as an allele in one amplicon, it could be classified as an artifact in another amplicon. At the conclusion of the workflow, we classified all variants as either an allele or an artifact.

In Steps IIIa & IIIb of the workflow, we classified variants into the following three categories: ‘1-2 bp differences’, ‘>2 bp differences’, and ‘chimera’ (Fig. 3). These classifications were based on the assumptions that, during PCR or sequencing, artifacts generated (1) occur less frequently than their parent allele, (2) occur less frequently than any non-parent true allele, and (3) are less likely than true alleles to appear in replicate PCRs. MHC alleles should be amplified in greater frequency than artifacts, although this may not always occur if the amplification efficiency of an allele is low relative to the artifact’s parent allele [19]. We calculated bp differences between variants using MEGA 5.2 [83] and identified potential chimeras using the *UCHIME de novo* command in UCHIME [89].

For the 255 individuals with replicate amplicons (*n* = 595 total amplicons, range = 1–6 replicates per individual), we examined variants within each amplicon first, then compared variant classifications between replicates for disagreement (Fig. 5: Step VI). Those variants with classification disagreements between replicates fell into the following two categories: (1) variants that were not labeled as an allele in any replicate (i.e., were labeled an artifact or an unclassified variant), which were then classified as artifacts for all replicates, or (2) variants that were labeled as an allele in at least one replicate, which became ‘low-efficiency alleles’ [19]. Some individuals (*n* = 47) were represented by only one successfully sequenced amplicon. Because we were unable to compare replicates for individuals, we modified our protocol to distinguish alleles from artifacts for those individuals (Fig. 3: Steps IIIb & IVb).

Lastly, owing to high coverage obtained for certain individuals, 34 lemurs initially had ≥ 16 alleles following workflow analysis. Their amplicons also contained no artifacts because, owing to the depth of coverage attained, none of the variants fulfilled the criteria for an artifact. Thus, all variants were initially classified as true alleles by default. These individuals appeared to have as many as eight MHC-*DRB* copies, whereas other individuals possessed a maximum of four MHC-*DRB* copies. Within these 34 individuals, many variants initially labeled as alleles were present only in those individuals that lacked any artifacts. We therefore classified these variants as artifacts. Any variant classified as an allele in another individual was retained as an allele. After this step, all genotyped lemurs had ≥ 8 alleles each.

After the initial analysis, our results clearly indicated that ring-tailed lemurs have > 1 MHC-*DRB* copies, contrary to our initial assumption. Our threshold of reads required to reliably genotype an individual therefore increased to 120 reads per individual [19, 27]. To confirm that at least 120 reads were required for reliable genotyping, we re-genotyped a subset of individuals using two alternative thresholds of (a) > 60 reads per amplicon and (b) > 200 reads per amplicon. Based on the minimum threshold of > 120 reads, we then re-genotyped any individuals for which any replicates fell below the threshold of 120 reads per amplicon, excluding amplicons with < 120 reads. We then named alleles in convention with previously established standards [90].

As a final verification of our genotyping protocol, we compared genotypes generated by our NGS data to predicted genotypes based on captive pedigree data, using 28 known parent-offspring trios from the historical DLC records. To evaluate NGS performance against traditional cloning, we compared genotypes obtained by NGS to genotypes obtained by cloning for 18 lemurs cloned at MHC-DRB.

After MHC-*DRB* genotypes were obtained for all individuals, we calculated dN/dS ratios for the entire MHC-*DRB* fragment, and the ABS and non-ABS regions separately, using MEGA 5.2 [83]. To test for positive selection on the second exon of MHC-*DRB*, we analyzed our alleles using Models 0, 1a, 2a, 7, and 8 in CODEML [91]. Lastly, we tested for trans-species polymorphism by constructing a phylogenetic tree, which included our new allelic sequences and all published lemur MHC-*DRB* sequences [46, 92].

## Results

**Fig. 6:**
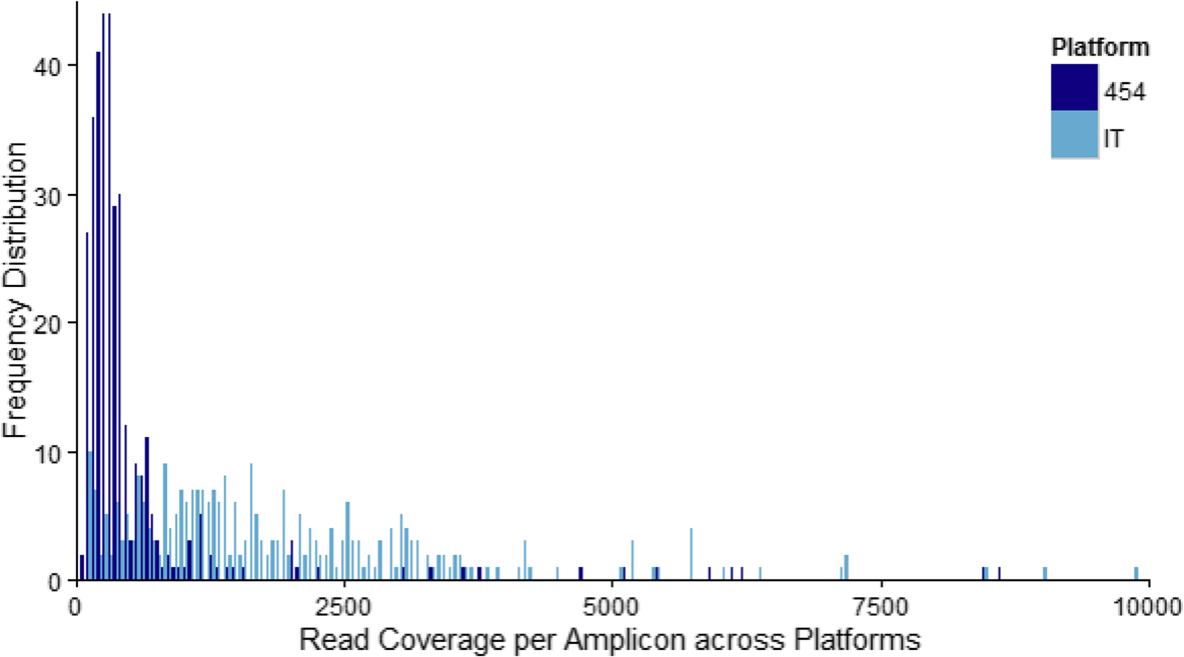
Frequency distribution of final read coverage per amplicons. The read coverage for each of the two sequencing platforms is shown: 454 Titanium [dark blue] and Ion Torrent PGM [light blue]. Because few amplicons had > 10,000 reads [*n* = 7], only the distribution of amplicons with < 10,000 reads are shown

In total, we generated 2,716,290 reads across the two NGS platforms, of which 1,113,646 (41.0 %) were retained after length and quality filtering on Galaxy (Table 1). These reads clustered into 48,920 unique variants. To examine differences between the platforms, we sequenced all individuals on each platform at least once. The platforms differed in the amount of data generated: the 454 Titanium 1/8^th^ lane produced an average coverage of 630 reads per amplicon, whereas the Ion Torrent PGM averaged 1,944 reads per amplicon (Fig. 6).

**Fig. 7:**
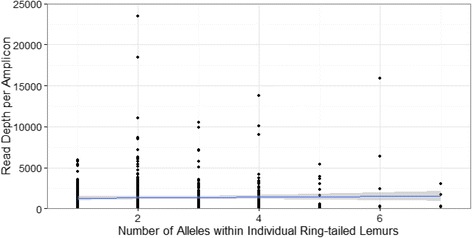
Relationship between the number of alleles per ring-tailed lemur and average read coverage per individual. Lack of correlation indicates that individuals were not under-sampled [Slope = 0.000, R^2^ = 0.001, *p* = 0.000]. Read coverage per individual was averaged across replicates for each individual and gray shading reflects 95 % confidence intervals

**Fig. 8:**
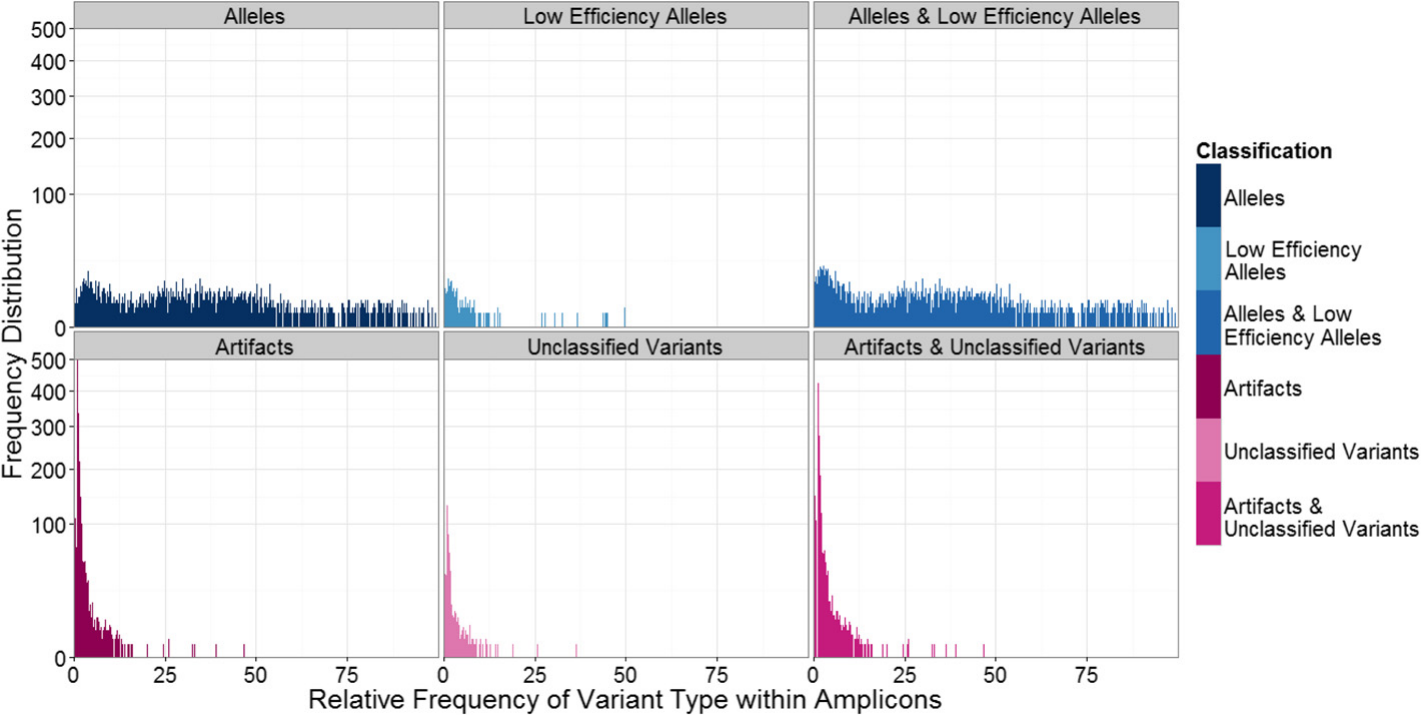
Frequency distribution for final variant classifications within amplicons. The top left and center graphs show alleles [dark blue] and low efficiency alleles [light blue], whereas the bottom left and center graphs display artifacts [dark pink] or unclassified variants [light pink]. The top right graph shows the combined relative frequencies alleles and low frequency alleles, and the bottom right graph shows the combined relative frequencies of artifacts and unclassified variants. The Y-axis is scaled as a square root

Using our workflow, we successfully genotyped 302 individuals from 642 amplicons combined across both NGS platforms. A minimum threshold of 120 reads per individual was sufficient for genotyping. We found that genotypes obtained using a minimum threshold of 60 reads differed from genotypes obtained using a threshold of 120 reads; however, genotyping using a minimum threshold of 200 reads produced identical genotypes to those obtained using a threshold of 120 reads. We were unable to genotype 18 individuals, due to insufficient read coverage. By comparing the number of alleles within an individual to the read coverage per individual [30, 51], we verified that we had not under-sequenced individuals because the number of alleles per individual was uncorrelated to the read depth per amplicon (Slope = 0.00002, R^2^ = 0.001, *p* = 0.00; Fig. 7). By the end of our workflow, we classified 52 variants as alleles, 36 variants as low-efficiency alleles, 188 variants as unclassified variants, and 1,008 variants as artifacts. The maximum relative frequency of all final ‘error’ classifications (i.e., artifacts and unclassified variants) overlapped the range of the relative frequency of alleles between 0.1 and 10 % (Fig. 8); however, in contrast to the 2.1 % average frequency of an artifact (range = 0.01-46.7 % of amplicon sequences) or the 1.94 % average frequency of an unclassified variant (range = 0.06-36.6 % of amplicon sequences), the average frequency of an allele was 35.1 % (range = 0.2-100.0 % of amplicon sequences), calculated across individuals possessing one to seven alleles. Of 1,079 unique variants spread among 642 amplicons, the classifications of only 156 variants disagreed. The majority (*n* = 109) of the disagreeing variants resulted in the classification combination of “artifact/unclassified variant”; the remaining 47 variants were classified in various combinations of alleles, low efficiencies, artifacts, and unclassified variants. We then collapsed the categories of ‘alleles’ and ‘low efficiency alleles’ into ‘alleles’, and unclassified variants into artifacts. We found no disagreement between the genotypes of samples obtained before and after whole genome amplification. Using historical records from the Duke Lemur Center to examine the genotypes of 28 known parent-offspring trios, we found agreement between 15 parent-offspring genotypes, whereas 13 offspring possessed at least one allele that was not present in either parent. Because these 13 offspring possessed either a unique genotype or possessed a ‘common’ genotype found in many other individuals in this study, sample contamination or mis-labeling of samples was unlikely. Lastly, we confirmed all NGS genotypes of 18 individuals, using traditional cloning (Additional file 1: Table S2). From these 18 individuals, six alleles were confirmed as true positives and all 18 genotypes showed 100 % congruence with genotypes assigned using NGS.

From 302 ring-tailed lemur genotypes, we found 55 unique putative MHC-*DRB* alleles (Additional file 1: Table S3), and identified the first sequences from this species for the MHC genes *DOA*, *DOB*, *DPA*, *DQA*, and *DRA* (Additional file 1: Table S4). Because we found only one allele each for *DOB*, *DPA*, *DQA*, and *DRA*, and two alleles for *DOA*, the diversity at these MHC genes appears to be far less than that present in the MHC-*DRB* gene. Individual lemurs possessed between 1 and 7 MHC-*DRB* alleles.

**Fig. 9:**
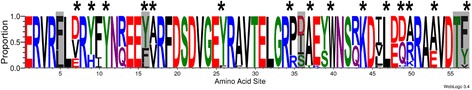
Sequence logo. This sequence logo [116] showing amino acid [AA] variation along the second exon of the ring-tailed lemur MHC-*DRB* gene. Letters represent standard AA abbreviations, letter height indicates the relative frequency in the population of each AA at that position, and letter color indicates the chemical properties of the AA: polar [green], neutral [purple], basic [blue], acidic [red], and hydrophobic [black]. * Identifies antigen-binding sites, assuming consensus with human *DRB* [117]. Gray boxes indicate positively selected sites calculated by models M2a & M8 in program PAML (Table 3: [91])

**Fig. 10:**
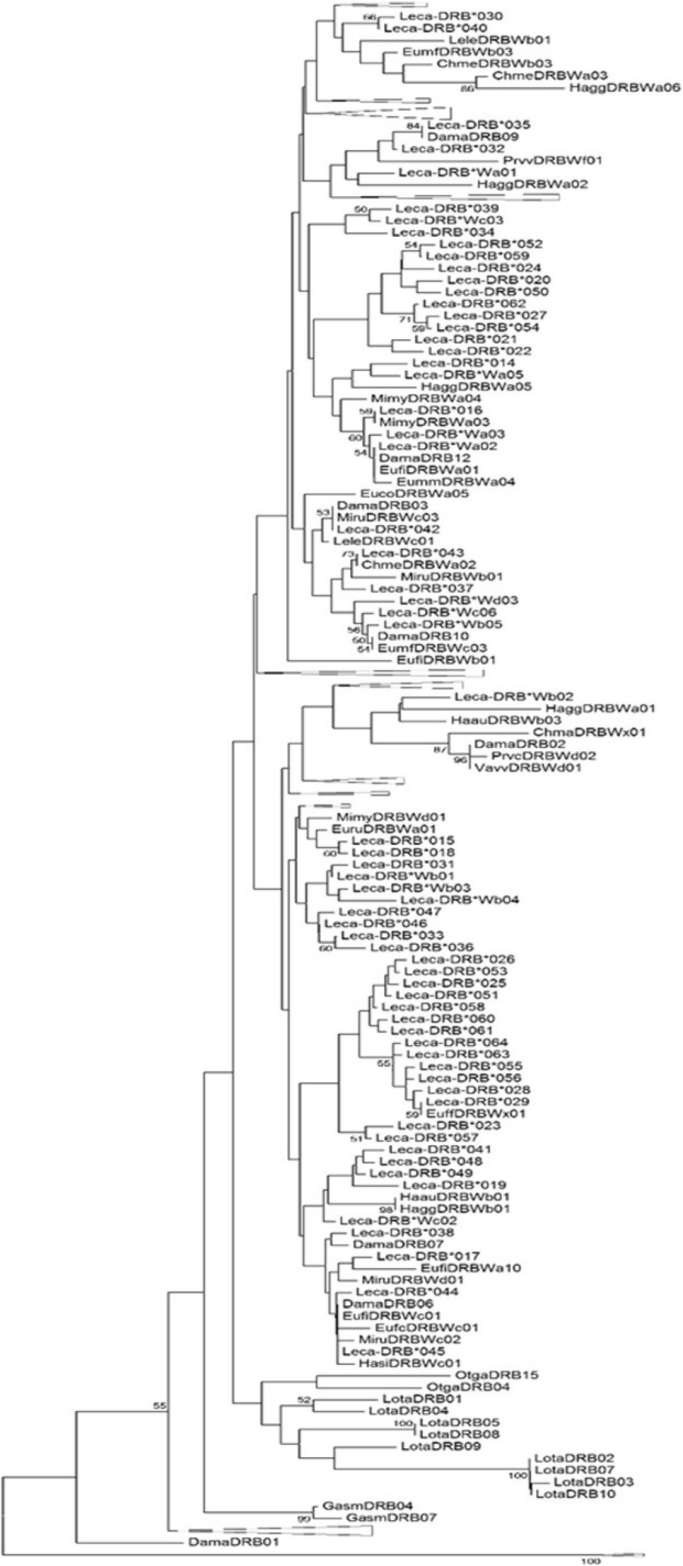
Phylogenetic tree of all ring-tailed lemur and all other published lemur MHC-*DRB* sequences. Ring-tailed lemur MHC-*DRB* sequences were produced by this study or downloaded from Genbank [92]. The phylogeny was created via neighbor-joining method with Kimura 2-Parameter [83, 118]. Species are designated by the first two letters of the genus and species names, e.g., ring-tailed lemur, or *Lemur catta*, sequences are indicated by 'Leca'. Triangles indicate collapsed nodes that did not contain ring-tailed lemur sequences. Bootstrap values > 50 are shown from 1000 replicates

Within the 55 MHC-*DRB* alleles, we found variation at 17.5 % of 171 nucleotide sites and at 36.8 % of 57 amino acid (AA) sites (Table 2, Fig. 9 & Additional file 1: S3). Between alleles, there was an average of 11.46 ± 3.07 nucleotide differences (range = 1–24) and 8.86 ± 2.64 AA differences (range = 1–17). Differences in AA sequence were concentrated at the antigen binding sites (ABS) and at positively selected sites (Fig. 9) and sequences showed a high dN/dS ratio along the entire sequence, the ABS sites, and the non-ABS sites (Table 2). Models M2a and M8 (that include positive selection) fit the data better than models 0, 1a, and 7 (that are limited to neutral selection: Table 3). Lastly, a phylogeny of all strepsirrhine MHC-*DRB* sequences showed no concordance with the lemur species tree (Fig. 10; [46, 92, 93]).

**Table 2:** Variation in amino acid sequence and rates of synonymous and nonsynonymous mutations across antigen and non-antigen binding MHC-*DRB* sites

Region of MHC-*DRB*	Total AA	Variable AA [%]	dAA ± S.E.	dN ± S.E.	dS ± S.E.	Z [*p*]
All sites	57	21 [36.8 %]	8.857 ± 2.65	0.090 ± 0.03	0.018 ± 0.02	3.985 [0.000*]
non-ABS sites	42	10 [23.8 %]	4.142 ± 1.88	0.051 ± 0.02	0.001 ± 0.00	2.745 [0.003*]
ABS sites	15	11 [73.3 %]	4.715 ± 1.66	0.217 ± 0.10	0.068 ± 0.07	2.767 [0.003*]

Note. This table shows the total number of amino acid codons [AA] as well as the number and percentage of variable amino acid codons [Variable AA] in the MHC-*DRB* second exon, the average [± S.E.] pairwise AA differences [dAA] between alleles, the average [± S.E.] rates of nonsynonymous [dN] and synonymous [dS] substitutions per site, and the one-tailed test of positive selection [Z] with *p*-values [*p*]. All standard errors were obtained from 1000 bootstrap replicates [83]

**Table 3:** Best fit of codon-based models of evolution to ring-tailed lemur MHC-*DRB* variation

MHC-*DRB*	LnL	AIC	ΔAIC	Parameters	PSS
M0 - one ratio [ω]	−1464.19	3184.38	378.64	ω = 0.60	Not applicable
M1a - neutral	−1302.65	2863.3	57.56	p0 = 0.65, ω0 = 0.0	Not applicable
M2a - selection	−1271.87	2805.74	0	p0 = 0.64, ω0 = 0.01, p1 = 0.25, ω1 = 1.0, p2 = 0.11, ω2 = 3.94	5, 16, 36, 56, 57
M7 - nearly neutral with β	−1298.41	2854.82	49.08	*p* = 0.05, q = 0.15	Not applicable
M8 - positive selection with β	−1273.31	2808.62	2.88	p0 = 0.87, p1 = 0.13, *p* = 0.02, q = 0.05, ω = 3.84	5, 16, 36, 56, 57

Note. Fit of data compared between models of strict neutral evolution [M0, M1a, & M7] and models including positive selection (M2a & M8; [91]). Fit assessed by log likelihood score [LnL], and Akaike information criteria [AIC] values; the model with the lowest AIC value, or a ΔAIC of zero, indicates the model that best fits the data. Estimated proportions of sites [p_X_] evolving at corresponding estimated rates [ω_X_ = dN/dS] are given in the parameters column, as well as positively selected sites [PSS], or AA sites under positive selection in each model

## Discussion

For hypervariable, multilocus genes like MHC-*DRB*, NGS is the preferred genotyping method, especially for large numbers of individuals [12, 19, 21, 27, 29–31, 52, 55, 56, 65, 94]. In this paper, we improved previously published methods for distinguishing alleles from artifacts, including additional steps to improve the detection of artifacts and allelic dropout [19]. Using our roadmap, we were able to genotype 94.6 % of our individuals with less expense and effort [45, 95], as well as greater depth of sequencing, than possible using previous ‘gold standard’ methods (e.g., cloning). The lack of correlation between read depth and number of alleles per individual indicates that we sequenced individuals at sufficient depth to avoid under-sampling and potentially missing alleles because of low coverage [65, 96]. Using a combination of gold-standard cloning and NGS techniques, we produced new sequence data for five previously unexplored MHC genes in the endangered ring-tailed lemur and confirmed that the MHC-*DRB* is by far the most variable of the six MHC genes investigated. We determined ring-tailed lemurs have at least four putatively functional copies of the MHC-*DRB* gene, consistent with the number of copies found in other primates ([46–48, 68], but see [12, 67]). Lastly, we demonstrated that the MHC-*DRB* gene in ring-tailed lemurs has experienced positive selective pressure in the past, supporting its importance in immune function.

Because sequencing depth depends on copy number, number of amplicons, and the number of reads that fail to pass length and quality filters [12, 19, 21, 27, 30, 31, 52, 56, 65, 94], researchers must plan amplicons pools carefully; otherwise, amplicons will lack sufficient coverage. As Roche ends technical support for all 454 machines in 2016, researchers will need to transition to other platforms like the Illumina MiSeq or Ion Torrent PGM. Based on the results of this study, researchers could pool as many as 400–500 individuals with four MHC-*DRB* copies on a single Ion Torrent PGM run (or even more individuals in species that have fewer gene copies).

In contrast to the well-developed methods for cloning or conformation-based genotyping, researchers are still refining protocols for NGS projects (reviewed in [65]). Methodologies are being modified and improved as new pitfalls are discovered. More specifically, the immense quantity of sequence data produced by NGS requires improved study designs. An important lesson from our study is that depth of sequencing is not a substitute for independent replicate PCRs. We highlight the need for increased sample replication between runs and platforms. We also suggest that each sample be sequenced from at least two independent PCRs, consistent with protocols in traditional cloning (e.g., [60]). Replicating only a few samples can increase the probability of obtaining the same genotype for each replicate by chance alone. For example, Lichten and colleagues [65] assigned genotypes for seven replicates with 100 % repeatability within a run; however, when the authors tested genotype repeatability across platforms by re-sequencing 49 samples on a different platform, genotype repeatability decreased to 83.7 % (see also [55, 61]). To date, the majority of researchers replicate ≤ 10 % of their sample populations using NGS, often within the same run and/or on the same platform (e.g., [21, 27, 28, 31, 52, 58, 61, 97, 98]). In studies in which < 10 % of samples are replicated, genotyping reliability is typically near 100 %; however, genotyping repeatability decreases as the amount of replication increases [30]. As sequencing costs decrease and we continue perfecting NGS methods, sequencing each sample from two independent PCR reactions should become standard protocol [59, 60, 99]. To achieve > 90 % genotyping success, we recommend researchers use a greater minimum coverage threshold to calculate their amplicon pools (e.g., [54]). This increased threshold accounts for both the loss of data during processing and for differing amplicon efficiencies.

Additionally, genotyping larger numbers of samples at greater depth can create unforeseen difficulties with the allele-calling workflow. For example, when samples are deep sequenced, some amplicons may be sequenced at such depth that even artifacts occur at great enough frequency to be classified as alleles using published allele-calling workflows [65]. In these cases, all amplicons variants are classified as alleles. To avoid this error, researchers should begin with an idea of copy number variation of MHC-*DRB* genes present in the study species and verify all genotypes using replication. Previous researchers have focused on low amplicons coverage impeding successful genotyping; however, as the quantity of data generated increases, we will likely discover new issues arising from greater sequence coverage of individuals.

Our final verification of genotypes, using known offspring-parent trios, identified another known pitfall of PCR-based sequencing – allelic dropout, which occurs when alleles are not detected in individuals that biologically possess them because of differences in allele amplification efficiency [19, 60]. We suggest that the mismatches we detected between parent and offspring genotypes resulted from allelic dropout. The 13 offspring in our population had been sired by seven different mothers, but only three fathers, and whereas the mothers produced additional offspring whose genotype could be matched to those of both parents, the three fathers sired only those offspring involved in the mismatches. We could therefore identify the fathers as the parent with an incorrect genotype owing to allelic dropout. Alternative explanations include sample mislabeling, misidentification of parents, or mutation of alleles between the parent and offspring generations. Sample mislabeling is unlikely because blood samples were collected and DNA extracted at several time points and we checked all sample identification at each step. Because replicates also failed to amplify the ‘missing’ alleles, amplicon mis-labeling or incorrect barcode assignment cannot explain the mismatches. We are confident in the parental attribution of the offspring because the putative parents were the only sexually mature adults present at the time of conception. Lastly, the rate of mutation in MHC alleles between generations is low [100–102]. Thus, mutations cannot account for the spontaneous appearance of new alleles in multiple offspring. These same alleles exhibited low amplification efficiency in other individuals; thus allelic dropout is the most likely explanation for the parent-offspring genotype mismatches we observed and has been observed in other parent-offspring MHC genotypes [103].

Based on our sequencing results, it is clear that ring-tailed lemurs retain MHC diversity despite their endangered status and declining population [104, 105]. The diversity at their MHC-*DRB* loci is comparable to the diversity found in other primate species, both threatened [46, 92, 106] and non-threatened [12, 19, 47–50, 92, 107]. Likewise, the low diversity at MHC-*DOA*, MHC-*DOB*, MHC-*DPA*, MHC-*DQA*, and MHC-*DRA* genes in ring-tailed lemurs was similar to the few alleles found at MHC-*DQA*, MHC-*DQB*, and MHC-*DRA* in mouse lemurs [67]. These latter genes are variably diverse in other non-human primates [108]. For instance, DP and DQ loci are variable in humans and apes, but show only limited variation in New World monkeys and strepsirrhines [67, 80]. In contrast to other MHC loci, particularly the *DRB* locus, the *DRA* locus exhibits low variation in humans [16], but moderate polymorphism in macaques [67]. Ring-tailed lemurs appear to have limited polymorphism at all of these loci, although sampling of additional individuals will be required to confirm these patterns. Minimal diversity at other MHC class II genes, combined with greater diversity at the MHC-*DRB*, indicates that the MHC-*DRB* gene is the most suitable candidate for investigating the fitness consequences of lemur genetic variation in future studies.

Because we sequenced from DNA rather than RNA, we could not assess if all of the MHC alleles uncovered were expressed and functional. In spite of this limitation, no alleles possessed stop codons so we can presume functionality. Our primers, however, amplified only 171 bp of exon 2. Although these 171 bp included the most variable region of the gene, the placement of the primers excluded any variation in the flanking regions of exon two, as well as variation in additional MHC-*DRB* loci. Therefore, we cannot assume that all lemur MHC-*DRB* loci are equally functional or under equal selective pressure. We were also unable to specify which alleles were found at which MHC-*DRB* copy. Nevertheless, evidence of historical, positive selection on ring-tailed lemurs is shown by the nucleotide and AA diversity found in MHC-*DRB* exon two, coupled with the high copy number, increased rate of nonsynonymous-to-synonymous substitutions, and better fit of codon-based models of selection that include positive selection. Pathogens likely drive this selection, though sexual selection also may be important [23, 92, 109]. Notably, we found the greatest between-sequence differences at the antigen binding sites. Many of these AA differences alter the binding properties of the sequence. We suggest from this evidence that different MHC-*DRB* molecules likely recognize different subsets of pathogens. Evidence of functional diversity, both between alleles and within an individual, reflects the strength of selective pressure that is likely exerted by pathogens.

As further evidence of the importance of disease resistance to an individual’s fitness, lemur MHC-*DRB* sequences show trans-species polymorphism, a phenomenon in which identical or nearly identical alleles are present in distantly related species [46, 54, 70, 101, 110–115]. Ring-tailed lemur alleles cluster with alleles from closely related genera, such as *Eulemur* and *Hapalemur*, but also with alleles from more distantly related Malagasy primate taxa, like *Daubentoniidae* and *Indriidae*, which diverged from the *Lemuridae* ~60 MYA and 24–40 MYA, respectively [93]. Because of these phylogenetic relationships, we suggest that balancing selection likely maintained these alleles throughout the strepsirrhine radiation. All lemur MHC-*DRB* alleles, however, form a monophyletic group within the greater strepsirrhine tree, just as all strepsirrhine alleles form a monophyletic group within the primate MHC-*DRB* tree. This pattern suggests that all lemur MHC-*DRB* alleles evolved after the most recent common ancestor of lemurids arrived on Madagascar [46, 93].

## Conclusions

Here, we highlight that NGS has the potential to alleviate many of the logistical and financial constraints that previously prevented genotyping large populations of non-model species at hypervariable loci like those genes in the MHC. As NGS genotyping becomes increasingly adopted by researchers, we will continue to discover new pitfalls to be avoided, especially when tackling the problem of distinguishing artifacts from true alleles. We suggest abandoning the use of absolute thresholds of sequence frequencies for distinguishing between alleles and artifacts. We also stress that each sample should be sequenced in duplicate and the replicates compared, preferably on a second run and if possible on a second platform to account for any run-specific or platform-specific errors. Historically, genotyping problems have resulted from the lack of sufficient coverage. As the capacity for data generation increases from tens of reads per sample to thousands or tens of thousands of reads per sample, this problem is no longer the primary limitation. Indeed, ‘ultra-deep’ sequencing gives rise to a new set of problems that require additional steps of error detection. Given the rapid expansion of these technologies and their increasing usage, studies such as the one presented here are critical to the transition from 454 sequencing to other NGS technologies.

## Additional file

Additional file 1: Supplementary materials [119]. (DOCX 43 kb)
