# Supplementary material for: Next-generation genotyping of hypervariable loci in many individuals of a non-model species: technical and theoretical implications
Source: BMC Genomics. 2016 Mar 8;17:204. doi: 10.1186/s12864-016-2503-y (PMC4782575; doi:10.1186/s12864-016-2503-y)
Supplement: Supplementary file 1 — Supplementary materials [119]. (DOCX 43 kb) [file 12864_2016_2503_MOESM1_ESM.docx]

**SUPPLEMENTARY MATERIALS**

**SUPPLEMENTARY METHODS**

Following initial quality assessment by the 454 and Ion Torrent software, we filtered the original FASTQ files using the open-sourced, web-based platform Galaxy [84-86] under the following conditions (Figure 1a): We removed any sequences substantially shorter (>150 bp) or longer (>400 bp) than the expected length of 171 bp, plus primers and barcodes. We also removed any sequences in which fewer than 95% of the base pairs had a Phred quality score of below 20. Using the program jMHC, we assigned all reads to individual amplicons based on the unique forward and reverse barcode combination [87]. To be retained by jMHC, reads must contain a perfect match to the primers and tags, without N calls [51, 87]. We also used jMHC to remove barcode and primer sequences, leaving only the exon sequence. We aligned that sequence, without gaps, to published ring-tailed lemur MHC-DRB sequences, using Galaxy Clustalw [Genbank Accession numbers: AB078199, AB078201, AB078229, AB078247, AB078248, AB078265, AB078279, AB078287, AB078288, AB078292, AB078301, AB078303; 46]. We discarded all sequences with an alignment score of less than 80 as artifacts.

Because insertion or deletion errors (indels) in homopolymer areas are the most common type of error in 454 or Ion Torrent sequencing [3-4, 36, 40, 43-44, 84], we corrected indels manually to reflect the most common consensus sequence at that location [65]. Once potential indel errors were corrected, we discarded any sequences that did not match the correct reading frame of 171 bp ± 3 bp. We did not retain shifts in reading frames because MHC-DRB is a coding region.

We then modified a workflow developed by Sommer and colleagues [19] for assigning reads as either sequencing artifacts or true alleles (Figure 1). Within each amplicon, we used jMHC to condense identical reads into a single sequence, with accompanying information about the total number of reads per amplicon represented by that sequence (Figure 1b). Because the number of MHC-DRB copies present in ring-tailed lemurs has not been determined, we initially assumed this species had the minimum number of copies (i.e., one). We therefore discarded only amplicons containing fewer than the 25 reads as having too few reads to genotype accurately [19, 27-28]. Within each amplicon, we discarded all singletons as artifacts due to probable PCR or sequencing error. We then compared replicates of the same individual, and discarded any sequence that did not represent greater than 1% of the total proportion of reads in any replicate amplicons as an artifact [31]. We performed all subsequent steps independently on each amplicon. In addition, we performed each step on all amplicons before moving onto the next step of the workflow. We analyzed variants independently and did not assume each only had one classification; thus if a sequence was classified as an allele in one amplicon, it need not be an allele in all other amplicons. We sorted sequences according to within amplicon frequency and the most frequent sequence was identified as a ‘putative allele,’ if it was also present in any replicates of that individual.

We classified all remaining sequencing into the following three categories: ‘1-2 bp differences’, ‘>2 bp differences’, and ‘chimera’ for subsequent analysis. These classifications were based on the assumptions that artifacts generated during PCR or sequencing: (1) occur less frequently than their parent allele, (2) occur less frequently than any non-parent true allele, and (3) are less likely than true alleles to appear in replicate PCRs. True MHC alleles should be amplified in greater frequency than artifacts, although this may not always occur if amplification efficiency for some alleles is lower than that of artifacts or if the initial PCR material contains errors in the case of poor quality samples [19]. At the conclusion of the workflow, we had classified all sequences as either a true allele or an artifact. We used MEGA 5.2 to calculate base pair differences for each sequence in comparison to any more frequent sequences within that amplicon, allowing for pairwise deletions [83]. To identify potential chimeras, we analyzed all remaining sequences using the *UCHIME de novo* command with the default parameters in the analysis tool UCHIME [89]. If a sequence was labeled as a potential chimera, then all amplicons containing that sequences were analyzed independently in UCHIME. To be defined as a chimera, a sequence must be identified as a chimera in all amplicons. If a sequence was present in any replicates, and classified as a chimera in one or more amplicons but as an allele in others, it was labeled an unclassified variant.

For individuals with replicate amplicons (n=255), we examined sequences within each amplicon separately, and then compared the resulting sequence classifications for disagreement. We labelled sequences in the ‘1-2 bp differences’ category as artifacts if they were not present in all replicates from the same individual. If sequences classified as ‘1-2 bp differences’ were present in all replicates, we classified them as (a) an allele, if their frequency was greater than the most frequent artifact, or (b) as an unclassified variant, if their frequency was lower than or equal to that of any artifact. We checked sequences in the ‘>2 bp differences’ category for their presence in any replicates. If these sequences were present in the replicate, we then identified sequences of greater frequency than any artifacts within that amplicon as alleles, whereas those of lower than or equal frequency to the most frequent artifact became unclassified variants. If a sequence in the ‘>2 bp differences’ category was not present in any replicates, but was present as an allele or an unclassified variant in another individual, we classified that sequences as an unclassified variant; however if it was an artifact in another individual or not present in any other individuals, we classified it as an artifact. We repeated these steps until all sequences within each amplicon were classified as an allele, an artifact, or an unclassified variant.

Some individuals (n=47) were represented by only one successfully sequenced amplicon. Because we were unable to compare replicates for those singleton individuals, we modified our protocol to distinguish alleles from artifacts for those individuals. The most frequent sequence within each amplicon was designated as a true allele, whereas any sequence found only in that amplicon or classified as an artifact in all other amplicons was categorized as an artifact. As in the procedure for amplicons with replicates, if a sequence (a) differed from the most frequent sequence in the amplicon by 1-2 bp, (b) was present in other individuals as an allele, and (c) had a greater relative frequency than any artifact, it was classified as an allele. If its frequency was lower than or equal to any artifact within that amplicon, it was labelled as an unclassified variant. If a sequence had more than 2 bp differences, was classified as either an unclassified variant or an artifact in any other amplicon, and was more frequent than any artifacts within that amplicon, it was labeled an allele. If the sequence was less frequent or had equal frequency to the most frequent artifact, it was labelled an unclassified variant.

This workflow allowed sequences to be differentially classified as alleles, unclassified variants, or artifacts in different amplicons. Within an individual, we checked the classification of each sequence across replicates for agreement. Of those sequences that disagreed in classification between replicates, they fell into the following two categories: (1) those sequences that were not labeled as an allele in any replicates, which were then classified as artifacts for all replicates, or (2) sequences which were labelled as allele in at least one replicate, which became ‘low efficiency alleles’ [19].

**Supplemental Table S1.** Forward and reverse primers developed for the genes MHC-DOA, MHC-DOB, MHC-DPA, MHC-DQA, MHC-DRA, and MHC-DRB [81].

| Gene | Forward Primer | Reverse Primer |
| --- | --- | --- |
| MHC-DOA exon 1 | 5’-GGACCAGCCTTCTACCAGTC-3’ | 5’-GTTGGAGCGTTCCACCAG-3’ |
| MHC-DOB exon 2 | 5’-CAGAGGTGACAGTGTACCCAGA-3’ | 5’-AGGCTGGGATGATCAACAA-3’ |
| MHC-DPA exon 2 | 5’-GAGGTGACTGTGTTTCCCAAG-3’ | 5’-AGGGCACGAAGGTCAGGTAG-3’ |
| MHC-DQA exon 2 | 5’-CCCAACACCCTCATCTGTCT-3’ | 5’-CAGTGCTCCACCTTGCAGT-3’ |
| MHC-DRA exon 1 | 5’-GATCATCCAGGCCGAGTTC-3’ | 5’-ATGATGTCCAGGTTGGCTTT-3’ |
| MHC-DRB exon 2 | 5’-GAGTGTCATTTCTWCAACGGGACG-3’ | 5’-GATCCCGTAGTTGTGTCTGCA-3’ |

**Supplemental Table S2.** Error rates of traditional cloning of MHC-DRB alleles from 18 individuals.

|  | N | Mean ± SD of clones with true alleles | Range of clones with true alleles | Mean ± SD of clones with artifacts | Range of clones with artifacts |
| --- | --- | --- | --- | --- | --- |
| Individuals with 1 MHC-DRB allele | 3 | 84.7% ± 4.5 | 80.3% - 89.3% | 15.3% ± 4.5 | 10.7% - 19.7% |
| Individuals with 2 MHC-DRB allele | 15 | 90.0% ± 7.5 | 77.2% - 100.0% | 10.0% ± 7.5 | 0.0% - 22.7% |

**Supplemental Table S3.** Ring-tailed lemur MHC-DRB exon 2 amino acid (AA) variation. Dots indicate identity with the top sequence whereas letters show variation. Variable positions are indicated by gray shading, and (*) identify ABS positions assuming consensus with humans [117]. The first 13 sequences were published in Genbank [46]; all other sequences were produced by this study. Genbank accession numbers are provided.

| Allele | Accession Number | ......*.*.*....**........*........*.*..*...*..*.**..*...* |
| --- | --- | --- |
| Leca-DRB*Wa01  Leca-DRB*Wa02  Leca-DRB*Wa03  Leca-DRB*Wa05  Leca-DRB*Wb01  Leca-DRB*Wb02  Leca-DRB*Wb03  Leca-DRB*Wb04  Leca-DRB*Wb05  Leca-DRB*Wc02  Leca-DRB*Wc03  Leca-DRB*Wc06  Leca-DRB*Wd03  Leca-DRB*014  Leca-DRB*015  Leca-DRB*016  Leca-DRB*017  Leca-DRB*018  Leca-DRB*019  Leca-DRB*020  Leca-DRB*021  Leca-DRB*022  Leca-DRB*023  Leca-DRB*024  Leca-DRB*025  Leca-DRB*026  Leca-DRB*027  Leca-DRB*028  Leca-DRB*029  Leca-DRB*030  Leca-DRB*031  Leca-DRB*032  Leca-DRB*033  Leca-DRB*034  Leca-DRB*035  Leca-DRB*036  Leca-DRB*037  Leca-DRB*038  Leca-DRB*039  Leca-DRB*040  Leca-DRB*041  Leca-DRB*042  Leca-DRB*043  Leca-DRB*044  Leca-DRB*045  Leca-DRB*046  Leca-DRB*047  Leca-DRB*048  Leca-DRB*049  Leca-DRB*050  Leca-DRB*051  Leca-DRB*052  Leca-DRB*053  Leca-DRB*054  Leca-DRB*055  Leca-DRB*056  Leca-DRB*057  Leca-DRB*058  Leca-DRB*059  Leca-DRB*060  Leca-DRB*061  Leca-DRB*062  Leca-DRB*063  Leca-DRB*064 | **AB078265**  **AB078287**  **AB078288**  **AB078292**  **AB078199**  **AB078229**  **AB078247**  **AB078248**  **AB078301**  **AB078201**  **AB078279**  **AB078303**  **AB078302**  **KJ817213**  **KJ817214**  **KJ817215**  **KJ817216**  **KJ817217**  **KJ817218**  **KJ817219**  **KJ817220**  **KJ817221**  **KJ817222**  **KJ817223**  **KJ817224**  **KJ817225**  **KJ817226**  **KJ817227**  **KJ817228**  **KJ817229**  **KJ817230**  **KJ817231**  **KJ817232**  **KJ817233**  **KJ817234**  **KJ817235**  **KJ817236**  **KJ817237**  **KJ817238**  **KJ817239**  **KJ817240**  **KJ817241**  **KJ817242**  **KJ817243**  **KJ817244**  **KJ817245**  **KJ817246**  **KJ817247**  **KJ817248**  **KJ817249**  **KJ817250**  **KJ817251**  **KJ817252**  **KJ817253**  **KJ817254**  **KJ817255**  **KJ817256**  **KJ817257**  **KJ817258**  **KJ817259**  **KJ817260**  **KJ817261**  **KJ817262**  **KJ817263** | ERVRFLDRYIYNREEYVRFDSDVGEYRAVTELGRPIAESWNSQKDFLEQKRAEVDTV  ....L.E......................................I...R..A...Y  ....L.E......................................I..HR..A...Y  ....L.E........F..................RS..Y...R..I...R......F  .........F......A..................D..Y...R..I.DDA..A...F  ....L....F......A........F.P.......D..Y......I.DYL..A....  .........F......A..................D..Y...R..I.DDA......F  .........F......A..................N..Y...R..I.DHT......F  ....L....F.....F....................TK.......I......A...F  .........F.....F..................RS..Y......I.DDA..A...F  .........F.....F..........................R..L...R......F  .........F.....F....................TK.......I......A...Y  ......E..F.....F....................TK.......I...R..A...Y  ....L.E........L......................Y...R..L...R......F  ....L.E............................D..Y...R..I.DDA..A...F  ....L.E........F.............................I...R..A...Y  ....L.E........F.............................I.DDA..A...F  ....L.E........F...................D..Y...R..I.DDA..A...F  ....L.E........F..................RS..Y...R..V.DDA..A...Y  ....L.V.HF..Q...A.....................Y...R..L...R......Y  ....L.V.HF..Q...A............................I......A...F  ....L.V.HF..Q...A............................I...R..A...Y  ....L.V.HF..Q...A............................I.DDA..A...F  ....L.V.HF..Q...A..................D..Y......L...R......Y  ....L.V.HF..Q...A..................D..Y...R..I.DDA..A...F  ....L.V.HF..Q...A..................D..Y...R..V.DDA..A...F  ....L.V.HF..Q...A.................RS..Y......I...R......F  ....L.V.HF..Q...A.................RS..Y...R..V.DDA..A...Y  ....L.V.HF..Q...A.................RS..Y...R..V.DDA..A...F  .........F..Q...A..................D..Y......L...R......Y  .........F..Q...A..................D..Y...R..I.DDA..A...F  .........F.....L...................D..Y..................  .........F.....L...................D..Y...R..I.DDA..A...F  .........F.....L...................D..Y...R..L...R......F  .........F.....L.........F.P.......D..Y..................  .........F.....L.........F.P.......D..Y...R..I.DDA..A...F  .........F......A............................I...R..A...Y  .........F......A............................I.DDA..A...F  .........F......A.........................R..L...R......F  .........F......A..................D..Y......L...R......Y  .........F......A.................RS..Y...R..V.DDA..A...Y  .........F.....F.............................I......A...F  .........F.....F.............................I...R..A...Y  .........F.....F.............................I.DDA..A...Y  .........F.....F.............................I.DDA..A...F  .........F.....F...................D..Y...R..I.DDA..A...F  .........F.....F...................D..Y...R..V.DDA..A...F  .........F.....F..................RS..Y...R..V.DDA..A...Y  .........F.....F..................RS..Y...R..V.DDA..A...F  ......V.HF..Q...A.........................R..L...R......F  ......V.HF..Q...A..................D..Y...R..I.DDA..A...F  ......V.HF..Q...A..................D..Y...R..L...R......F  ......V.HF..Q...A..................D..Y...R..V.DDA..A...F  ......V.HF..Q...A.................RS..Y......I...R......F  ......V.HF..Q...A.................RS..Y...R..V.DDA..A...Y  ......V.HF..Q...A.................RS..Y...R..V.DDA..A...F  ......V..F..Q...A............................I.DDA..A...F  ......V..F..Q...A..................D..Y...R..I.DDA..A...F  ......V..F..Q...A..................D..Y...R..L...R......F  ......V..F..Q...A..................D..Y...R..V.DDA..A...Y  ......V..F..Q...A..................D..Y...R..V.DDA..A...F  ......V..F..Q...A.................RS..Y......I...R......F  ......V..F..Q...A.................RS..Y...R..V.DDA..A...Y  ......V..F..Q...A.................RS..Y...R..V.DDA..A...F |

**Supplemental Table S4.** Amino acid (AA) variation of MHC-DOA, MHC-DOB, MHC-DPA, MHC-DQA, and MHC-DRA genes in ring-tailed lemurs compared to sequences from the mouse lemur (Mmur), the bushbaby (Ogar), and Tarsier (Tsyr) downloaded from IGMT/HLA databases [16] and Ensembl Genome Browser [119]. Dots indicate identity with the top sequence, letters indicate site variation, dashes (-) indicate sequence gaps, and (*) indicates a stop codon. Genbank accession numbers for all sequences are provided.

| Allele | Accession Number | AA Sequence |
| --- | --- | --- |
| Leca-DOA*01  Leca-DOA*02  Mmur-DOA  Ogar-DOA | **KJ786286**  **KJ786288** | YEFDGEQLFSVDLKKSEAVWRL-AFGDFAHFDPQ--LASIAMIRAHLDVLVERSN  ......................P...........GG...................  H.............ERQ.....PE.....Y....GG.N......D..........  ......................PE..N.......GGP..L............... |
| Leca-DOB*01  Tsyr-DOB  Ogar-DOB | **KJ817265** | QQHNLLLCSVTGFYPGDIKIRWFWNGQEERAGVVSTGLVRNGDWTFQTTVMLEMTPELGDVYTCLVDHPSL  .......................R......T.IM..D.I.........M.....I................  ..................N...LR.........L..D.I.........V...................... |
| Leca-DPA*01  Mmur-DPA  Ogar-DP | **LK391421** | ELGQPNTLICHIDKFFPPVLNATWL-NGQPVTEGVAESLFLPRTDYNFHKFHYLTFVP  .....................V...-......................C.........  ............N........V...-......Q..G...................... |
| Leca-DQA*01  Mmur-DQA  Ogar-DQA | **KJ817264** | FPPVINISWLSNGHSVTEGVSETSFLSKSDHSFLKISYLTFLPSADEIYDCKVEH*  ..........G...P..........I....................D........WGLDEPLLKHW  ............................................D.VV.................. |
| Leca-DRA*01  Mmur-DRA  Ogar-DRA | **LK391420** | EFMFDFDGDEIFHVDLDKKETVWRLEEFGRFASFEAQGALANIAVDKANLDIMIKRS  ...............M..R..................................M...  ...............MG............QY.G........................ |
